# Bis(creatininium) tetra­chlorido­cadmate(II)

**DOI:** 10.1107/S1600536809026944

**Published:** 2009-07-18

**Authors:** Sihem Boufas, Toma-Nardjes Mouas, Patricia Bénard-Rocherullé

**Affiliations:** a Université 20 Aout 1955, Skikda, Algeria; b Université Mentouri, Constantine, Algeria; c UMR 6226 CNRS Sciences Chimiques de Rennes, Université Rennes 1, Rennes, France

## Abstract

In the title compound, (C_4_H_8_N_3_O)_2_[CdCl_4_], the asymmetric unit comprises two creatininium cations and one tetra­chloridocadmate anion. Cd⋯O secondary bonding links one of the two imidazole rings and the anion into ion pairs. The free and bound cations form layers between which the [CdCl_4_]^2−^ anions are sandwiched. The Cd^II^ atom adopts a distorted trigonal-bipyramidal geometry in which the Cd⋯O bond is axial. Inter­molecular N—H⋯Cl hydrogen bonds form a two-dimensional network parallel to (001) which ensures the junction between creatininium cations and [CdCl_4_]^2−^ anions.

## Related literature

An abnormal level of creatinine in biological fluids is an indicator of various medical conditions, see: Narayanan & Appleton (1980 ▶). For inter­actions between creatinine and biologically important metal ions, see: Canty *et al.* (1979 ▶). Different complex species are formed depending on the reaction conditions, see: Nishida & Kida (1985 ▶). For bond lengths in the neutral creatinine mol­ecule, see: Smith & White (2001 ▶) and in creatinium compounds, see: Wilkinson & Harrison (2005 ▶). For Cd—Cl bond distances in dichlorido­bis(creatinine)cadmium(II), see: Okabe *et al.* (1995 ▶). For Cl—Cd—Cl bond angles in bis­(2,3,5-triphenyl­tetra­zolium)tetra­chloridocadmate(II), see: Zhang *et al.* (2007 ▶). For hydrogen-bond motifs, see: Bernstein *et al.* (1995 ▶); Etter *et al.* (1990 ▶). 
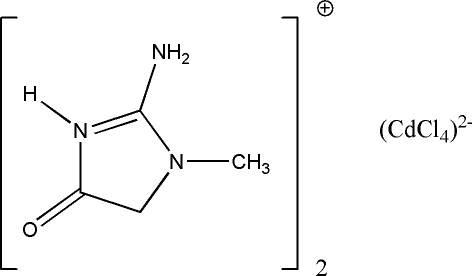


## Experimental

### 

#### Crystal data

(C_4_H_8_N_3_O)_2_[CdCl_4_]
*M* *_r_* = 482.48
Triclinic, 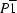

*a* = 7.5203 (4) Å
*b* = 7.6761 (3) Å
*c* = 15.0757 (7) Å
α = 79.476 (2)°
β = 85.438 (5)°
γ = 83.214 (3)°
*V* = 848.14 (7) Å^3^
*Z* = 2
Mo *K*α radiation
μ = 1.93 mm^−1^
*T* = 100 K
0.25 × 0.15 × 0.1 mm

#### Data collection

Nonius KappaCCD diffractometer
Absorption correction: multi-scan (*SADABS*; Sheldrick,1996 ▶) *T*_min_ = 0.644, *T*_max_ = 0.831
10950 measured reflections
3843 independent reflections
3711 reflections with *I* > 2σ(*I*)
*R*_int_ = 0.064

#### Refinement

*R*[*F* ^2^ > 2σ(*F* ^2^)] = 0.019
*wR*(*F* ^2^) = 0.048
*S* = 1.11
3843 reflections
216 parameters
H atoms treated by a mixture of independent and constrained refinement
Δρ_max_ = 0.77 e Å^−3^
Δρ_min_ = −0.54 e Å^−3^

### 

Data collection: *COLLECT* (Nonius, 2002 ▶); cell refinement: *DENZO* and *SCALEPACK* (Otwinowski & Minor, 1997 ▶); data reduction: *DENZO* and *SCALEPACK*; program(s) used to solve structure: *SIR92* (Altomare *et al.*, 1993 ▶); program(s) used to refine structure: *SHELXL97* (Sheldrick, 2008 ▶); molecular graphics: *ORTEP-3* (Farrugia, 1997 ▶) and *Mercury* (Macrae *et al.*, 2006 ▶); software used to prepare material for publication: *WinGX* (Farrugia, 1999 ▶) and *PARST* (Nardelli, 1995 ▶).

## Supplementary Material

Crystal structure: contains datablocks global, I. DOI: 10.1107/S1600536809026944/bt2978sup1.cif

Structure factors: contains datablocks I. DOI: 10.1107/S1600536809026944/bt2978Isup2.hkl

Additional supplementary materials:  crystallographic information; 3D view; checkCIF report

## Figures and Tables

**Table:** 

Cd1—Cl1	2.4571 (5)
Cd1—Cl4	2.4596 (4)
Cd1—Cl2	2.4627 (4)
Cd1—Cl3	2.5678 (4)
Cd1—O1	2.6854 (13)

**Table:** 

Cl1—Cd1—Cl4	116.512 (15)
Cl1—Cd1—Cl2	120.122 (17)
Cl4—Cd1—Cl2	117.621 (16)
Cl1—Cd1—Cl3	96.649 (15)
Cl4—Cd1—Cl3	98.074 (15)
Cl2—Cd1—Cl3	99.319 (15)
Cl1—Cd1—O1	82.18 (3)
Cl4—Cd1—O1	82.85 (3)
Cl2—Cd1—O1	80.95 (3)
Cl3—Cd1—O1	178.75 (3)

**Table 2:** Hydrogen-bond geometry (Å, °)

*D*—H⋯*A*	*D*—H	H⋯*A*	*D*⋯*A*	*D*—H⋯*A*
N1—H1⋯Cl3	0.79 (2)	2.41 (2)	3.1840 (16)	168 (2)
N2—H2⋯Cl3^i^	0.80 (3)	2.48 (3)	3.273 (2)	170 (3)
N4—H4⋯Cl4	0.75 (2)	2.70 (2)	3.2967 (17)	139 (2)
N6—H6⋯Cl2^ii^	0.88 (3)	2.32 (3)	3.1673 (18)	161 (3)
N2—H22⋯Cl1	0.90 (2)	2.34 (2)	3.2368 (18)	171 (2)
N6—H66⋯Cl4^iii^	0.81 (3)	2.55 (3)	3.2117 (17)	140 (2)

Symmetry codes: (i) 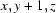; (ii) 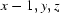; (iii) 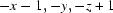.

**Fig. 1.**
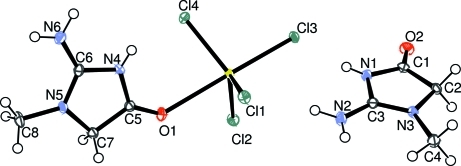
The independent components of (I), showing the atom-labelling scheme. Displacement ellipsoids are drawn at the 50% probability level.

**Fig. 2.**
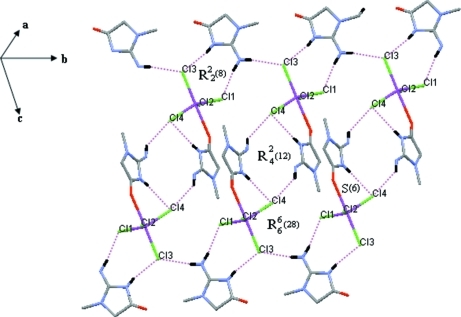
Part of the crystal structure of (I), showing the formation of the R22(8), R24(12) and R66(28) rings. Hydrogen bonds are shown as dashed lines. Hydrogen atoms not involved in the motif have been omitted for clarity.

**Fig. 3.**
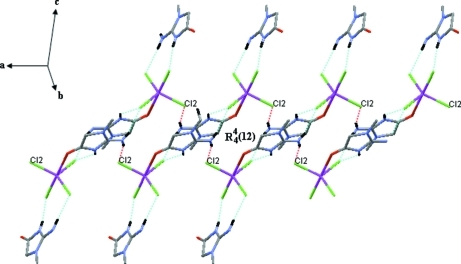
Part of the crystal structure of (I), showing the R44(12) ring set motif

## supplementary crystallographic information

### Comment

Creatinine (2-amino- 1,5-dihydro- 1-methyl-4*H*-imidazol-4-one) is the waste product of protein metabolism that is found in the urine. It can be measured to assess overall kidney function. An abnormal level of creatinine in biological fluids is an indicator of various malady states (Narayanan & Appleton, 1980).

Over the last decade, there have been an increasing number of reports on compounds resulting from the combination of creatinine and biologically important metal ions, in order to obtain structural information on the mode of interaction between them. (Canty *et al.*, 1979), however, the different complex species are formed depending on the reaction conditions (Nishida & Kida, 1985). In the title compound, (I), we determine the crystal structure of a new cadmium complex showing the metal coordination and the structure cohesion. It is the first example of salt containing creatininium cation and (CdCl_4_)^2-^ anion.

The asymmetric unit, shown in Fig. 1, consists of the tetrachlorocadmate anion and two cations of creatinine protonated. Hence, the complex investigated is better formulated as: 2(C_4_H_8_ON_3_)^+^,(CdC1_4_)^2-^.

The cadmium is coordinated to four Cl atoms and one O atom forming a distorted trigonal bipyramid Cd*X*_4_O. As can be seen in Table 1, three of the four Cd1—Cl bond distances (2.4571 (5), 2.4596 (4) and 2.4627 (4) Å) are similar to those found in the dichlorobis(creatinine)cadmium(II) (Okabe *et al.*, 1995) and significantly shorter than the axial Cd···Cl3 distance (2.5678 (4) Å) witch is in *trans* position to the O atom (d_Cd1—O_ = 2.6854 (13) Å and 0.1 Å longer than the other chlorine atoms. In the trigonal bipyramid CdCl4O, the displacement of Cd towards Cl3 from the equatorial plane defined by Cl1; Cl2 and Cl4 atoms is 0.2574 (2) Å. Furthermore, the Cl—Cd—Cl bond angles in the title complex fall in the range 96.649 (15) ° -120.122 (17) ° which is very different to those of the free anionic CdCl_4_ moiety of bis(2,3,5-triphenyltetrazolium) tetrachloridocadmate(II) (Zhang *et al.*, 2007) where the Cl—Cd—Cl bond angles lie in the range 107.18 (3) ° - 117.232 (16) °.

As expected, creatininium cations are approximately planar. In the bound cation, the r.m.s. deviation for the non-H atoms = 0.0311 Å, the maximum deviation of N5 from the mean plane is 0.0592 (15) Å. However, the free cation has a r.m.s deviation for non-H atoms = 0.0488 Å with a maximum deviation from the mean plane = 0.0851 (0.11) Å (for O2).

In table 1, we notice that in each creatininium cation, the three C—N bond distances are clearly different, with C6—N4 and C3—N1 much longer than the other ones. This results in the localization of the exocyclic C3—N2 [1.325 (2) Å] & C3—N3 [1.321 (2) Å] and C6—N5 [1.319 (2) Å] & C6—N6 [1.322 (2) Å] double bonds in the free and bound creatininium respectively and the adjacent single bonds C3—N1 [1.371 (2) Å] and C6—N4 [1.380 (2) Å]. These values somewhat compared with the intermediate, delocalized values in the parent neutral creatinine molecule [1.320 (3) and 1.349 (3) Å], respectively (Smith & White, 2001). Such difference in C—N bonds has been reported in some creatinium compounds (Wilkinson *et al.*, 2005).

In the crystal structure of (I), the anionic and cationic components are linked by N—H···Cl, hydrogen bonds into a continuous two-dimensional network (Table 3). In which alternating *R*^2^_2_(8), *R*^2^_4_(12) and *R*^6^_6_(28) (Bernstein, *et al.*,1995) hydrogen-bonded rings are formed. Fig. 2 also shows a strong intramolecular hydrogen bond (N4—H4···Cl4) which can be described with an S(6) graph set motif. These rings form layers joined by means of the N6—H6···Cl2 hydrogen bond. In terms of graph-set analysis (Etter *et al.*, 1990; Bernstein *et al.*, 1995), the descriptor for this pattern is *R*^4^_4_(12) ring (Fig. 3).

### Experimental

The title compound was crystallized from a supersaturated hydrochloric acid solution (45%, 5 ml) prepared using doubly distilled water and a mixture of cadmium(II) chloride (1.83 g) and creatinine (2.26 g). Colourless plates-shaped single crystals of (I) were obtained at ambient temperature by slow evaporation of the solution.

### Refinement

The iminium and ammino H atoms were located in a difference Fourier map and was refined isotropically. The methyl H atoms were constrained to an ideal geometry (C—H = 0.96 Å) with *U*_iso_(H) = 1.2*U*_eq_(C), but were allowed to rotate freely about the C—C bonds. The methylene H atoms were placed in geometrically idealized positions (C—H = 0.97 Å) and constrained to ride on their parent atoms with *U*_iso_(H) = 1.2*U*_eq_(C).

### Crystal data

**Table:**

(C_4_H_8_N_3_O)_2_[CdCl_4_]	*Z* = 2
*M**_r_* = 482.48	*F*(000) = 476
Triclinic, *P*1	*D*_x_ = 1.889 Mg m^−^^3^
Hall symbol: -P 1	Mo *K*α radiation, λ = 0.71073 Å
*a* = 7.5203 (4) Å	Cell parameters from 8132 reflections
*b* = 7.6761 (3) Å	θ = 2.7–27.4°
*c* = 15.0757 (7) Å	µ = 1.93 mm^−^^1^
α = 79.476 (2)°	*T* = 100 K
β = 85.438 (5)°	Plates, colourless
γ = 83.214 (3)°	0.25 × 0.15 × 0.1 mm
*V* = 848.14 (7) Å^3^	

### Data collection

**Table:**

Nonius KappaCCD diffractometer	3843 independent reflections
Radiation source: fine-focus sealed X-ray tube	3711 reflections with *I* > 2σ(*I*)
graphite	*R*_int_ = 0.064
φ scans, and ω scans with κ offsets	θ_max_ = 27.4°, θ_min_ = 2.7°
Absorption correction: multi-scan (*SADABS*; Sheldrick,1996)	*h* = −9→9
*T*_min_ = 0.644, *T*_max_ = 0.831	*k* = −9→9
10950 measured reflections	*l* = −19→19

### Refinement

**Table:**

Refinement on *F*^2^	Secondary atom site location: difference Fourier map
Least-squares matrix: full	Hydrogen site location: inferred from neighbouring sites
*R*[*F*^2^ > 2σ(*F*^2^)] = 0.019	H atoms treated by a mixture of independent and constrained refinement
*wR*(*F*^2^) = 0.048	*w* = 1/[σ^2^(*F*_o_^2^) + (0.0154*P*)^2^ + 0.4426*P*] where *P* = (*F*_o_^2^ + 2*F*_c_^2^)/3
*S* = 1.11	(Δ/σ)_max_ = 0.002
3843 reflections	Δρ_max_ = 0.77 e Å^−^^3^
216 parameters	Δρ_min_ = −0.54 e Å^−^^3^
0 restraints	Extinction correction: *SHELXL97* (Sheldrick, 2008), Fc^*^=kFc[1+0.001xFc^2^λ^3^/sin(2θ)]^-1/4^
Primary atom site location: structure-invariant direct methods	Extinction coefficient: 0.08 (2)

### Special details

**Table:**

Geometry. All e.s.d.'s (except the e.s.d. in the dihedral angle between two l.s. planes) are estimated using the full covariance matrix. The cell e.s.d.'s are taken into account individually in the estimation of e.s.d.'s in distances, angles and torsion angles; correlations between e.s.d.'s in cell parameters are only used when they are defined by crystal symmetry. An approximate (isotropic) treatment of cell e.s.d.'s is used for estimating e.s.d.'s involving l.s. planes.

### Fractional atomic coordinates and isotropic or equivalent isotropic displacement parameters (Å^2^)

**Table:**

	*x*	*y*	*z*	*U*_iso_*/*U*_eq_	
Cd1	0.066723 (15)	0.225860 (16)	0.280951 (8)	0.01066 (5)	
Cl1	−0.04670 (6)	0.54257 (6)	0.24433 (3)	0.01616 (9)	
Cl2	0.36325 (5)	0.13754 (7)	0.34349 (3)	0.01814 (10)	
Cl3	0.14704 (6)	0.17937 (6)	0.11755 (3)	0.01524 (9)	
Cl4	−0.15980 (5)	0.01360 (6)	0.32005 (3)	0.01418 (9)	
O1	−0.01715 (17)	0.28215 (19)	0.45064 (9)	0.0176 (3)	
N4	−0.3242 (2)	0.2622 (2)	0.47014 (11)	0.0130 (3)	
N5	−0.38183 (19)	0.3218 (2)	0.60861 (10)	0.0134 (3)	
N6	−0.6224 (2)	0.2535 (2)	0.53371 (12)	0.0179 (3)	
C5	−0.1579 (2)	0.2895 (2)	0.49571 (12)	0.0127 (3)	
C6	−0.4526 (2)	0.2762 (2)	0.53994 (12)	0.0126 (3)	
C7	−0.1888 (2)	0.3305 (3)	0.59036 (12)	0.0144 (4)	
H7A	−0.1208	0.2427	0.633	0.017*	
H7B	−0.1565	0.4481	0.5929	0.017*	
C8	−0.4703 (3)	0.3343 (3)	0.69777 (13)	0.0213 (4)	
H8A	−0.4273	0.4292	0.7208	0.026*	
H8B	−0.444	0.2239	0.7383	0.026*	
H8C	−0.5976	0.358	0.6926	0.026*	
O2	0.40943 (18)	0.36919 (18)	−0.10195 (9)	0.0191 (3)	
N1	0.2474 (2)	0.5415 (2)	−0.00578 (11)	0.0139 (3)	
C1	0.3552 (2)	0.5126 (2)	−0.08243 (12)	0.0138 (3)	
C2	0.3922 (2)	0.6952 (2)	−0.13185 (12)	0.0127 (3)	
H2A	0.52	0.7038	−0.1423	0.015*	
H2B	0.3364	0.7239	−0.1893	0.015*	
N2	0.1430 (2)	0.7800 (2)	0.07081 (11)	0.0176 (3)	
N3	0.3113 (2)	0.8115 (2)	−0.06971 (10)	0.0129 (3)	
C3	0.2295 (2)	0.7185 (2)	0.00098 (12)	0.0130 (3)	
C4	0.3227 (3)	1.0033 (2)	−0.08903 (13)	0.0166 (4)	
H4A	0.2574	1.0553	−0.1413	0.02*	
H4B	0.4461	1.0256	−0.1004	0.02*	
H4C	0.2722	1.0551	−0.0381	0.02*	
H4	−0.340 (3)	0.224 (3)	0.4298 (17)	0.017 (6)*	
H66	−0.689 (4)	0.238 (3)	0.5785 (19)	0.032 (7)*	
H6	−0.653 (4)	0.215 (4)	0.486 (2)	0.038 (8)*	
H1	0.212 (3)	0.462 (3)	0.0292 (17)	0.024 (6)*	
H22	0.092 (3)	0.704 (3)	0.1156 (17)	0.024 (6)*	
H2	0.142 (3)	0.884 (4)	0.0753 (18)	0.030 (7)*	

### Atomic displacement parameters (Å^2^)

**Table:**

	*U*^11^	*U*^22^	*U*^33^	*U*^12^	*U*^13^	*U*^23^
Cd1	0.00992 (7)	0.01213 (8)	0.01001 (7)	−0.00125 (5)	−0.00016 (5)	−0.00229 (5)
Cl1	0.0200 (2)	0.0126 (2)	0.0151 (2)	0.00070 (16)	−0.00008 (16)	−0.00248 (16)
Cl2	0.01099 (18)	0.0271 (3)	0.0158 (2)	0.00115 (16)	−0.00235 (15)	−0.00372 (18)
Cl3	0.0220 (2)	0.0142 (2)	0.0106 (2)	−0.00484 (16)	0.00282 (16)	−0.00459 (16)
Cl4	0.01278 (18)	0.0140 (2)	0.0155 (2)	−0.00334 (15)	0.00140 (15)	−0.00161 (16)
O1	0.0146 (6)	0.0226 (7)	0.0159 (7)	−0.0034 (5)	0.0043 (5)	−0.0055 (5)
N4	0.0150 (7)	0.0162 (8)	0.0090 (8)	−0.0040 (6)	0.0002 (6)	−0.0044 (6)
N5	0.0122 (7)	0.0171 (8)	0.0110 (7)	−0.0009 (6)	0.0017 (5)	−0.0042 (6)
N6	0.0132 (7)	0.0249 (9)	0.0151 (8)	−0.0055 (6)	−0.0003 (6)	−0.0005 (7)
C5	0.0147 (8)	0.0111 (9)	0.0121 (9)	−0.0020 (6)	0.0001 (6)	−0.0013 (7)
C6	0.0144 (8)	0.0101 (8)	0.0120 (8)	−0.0014 (6)	0.0008 (6)	0.0009 (7)
C7	0.0130 (8)	0.0181 (9)	0.0129 (9)	−0.0014 (6)	0.0001 (6)	−0.0052 (7)
C8	0.0223 (9)	0.0278 (11)	0.0132 (9)	−0.0003 (8)	0.0066 (7)	−0.0072 (8)
O2	0.0242 (7)	0.0130 (7)	0.0206 (7)	0.0005 (5)	−0.0015 (5)	−0.0059 (5)
N1	0.0178 (7)	0.0111 (8)	0.0125 (8)	−0.0033 (6)	0.0006 (6)	−0.0009 (6)
C1	0.0137 (8)	0.0155 (9)	0.0130 (9)	−0.0013 (6)	−0.0040 (7)	−0.0032 (7)
C2	0.0148 (8)	0.0135 (9)	0.0102 (8)	−0.0009 (6)	0.0008 (6)	−0.0039 (7)
N2	0.0241 (8)	0.0143 (9)	0.0143 (8)	−0.0038 (6)	0.0052 (6)	−0.0039 (7)
N3	0.0170 (7)	0.0105 (8)	0.0116 (7)	−0.0018 (6)	0.0014 (6)	−0.0034 (6)
C3	0.0140 (8)	0.0125 (9)	0.0129 (9)	−0.0022 (6)	−0.0019 (6)	−0.0021 (7)
C4	0.0226 (9)	0.0115 (9)	0.0158 (9)	−0.0026 (7)	0.0017 (7)	−0.0031 (7)

### Geometric parameters (Å, °)

**Table:**

Cd1—Cl1	2.4571 (5)	C8—H8B	0.96
Cd1—Cl4	2.4596 (4)	C8—H8C	0.96
Cd1—Cl2	2.4627 (4)	O2—C1	1.208 (2)
Cd1—Cl3	2.5678 (4)	N1—C3	1.371 (2)
Cd1—O1	2.6854 (13)	N1—C1	1.394 (2)
O1—C5	1.213 (2)	N1—H1	0.79 (3)
N4—C6	1.380 (2)	C1—C2	1.508 (3)
N4—C5	1.387 (2)	C2—N3	1.460 (2)
N4—H4	0.75 (2)	C2—H2A	0.97
N5—C6	1.319 (2)	C2—H2B	0.97
N5—C7	1.463 (2)	N2—C3	1.325 (2)
N5—C8	1.466 (2)	N2—H22	0.90 (3)
N6—C6	1.322 (2)	N2—H2	0.81 (3)
N6—H66	0.81 (3)	N3—C3	1.321 (2)
N6—H6	0.87 (3)	N3—C4	1.459 (2)
C5—C7	1.511 (2)	C4—H4A	0.96
C7—H7A	0.97	C4—H4B	0.96
C7—H7B	0.97	C4—H4C	0.96
C8—H8A	0.96		
			
Cl1—Cd1—Cl4	116.512 (15)	N5—C8—H8B	109.5
Cl1—Cd1—Cl2	120.122 (17)	H8A—C8—H8B	109.5
Cl4—Cd1—Cl2	117.621 (16)	N5—C8—H8C	109.5
Cl1—Cd1—Cl3	96.649 (15)	H8A—C8—H8C	109.5
Cl4—Cd1—Cl3	98.074 (15)	H8B—C8—H8C	109.5
Cl2—Cd1—Cl3	99.319 (15)	C3—N1—C1	110.39 (15)
Cl1—Cd1—O1	82.18 (3)	C3—N1—H1	127.6 (18)
Cl4—Cd1—O1	82.85 (3)	C1—N1—H1	121.8 (18)
Cl2—Cd1—O1	80.95 (3)	O2—C1—N1	125.86 (18)
Cl3—Cd1—O1	178.75 (3)	O2—C1—C2	128.57 (17)
C5—O1—Cd1	132.55 (13)	N1—C1—C2	105.55 (15)
C6—N4—C5	110.28 (15)	N3—C2—C1	102.80 (14)
C6—N4—H4	122.8 (18)	N3—C2—H2A	111.2
C5—N4—H4	125.3 (18)	C1—C2—H2A	111.2
C6—N5—C7	110.56 (14)	N3—C2—H2B	111.2
C6—N5—C8	126.70 (15)	C1—C2—H2B	111.2
C7—N5—C8	122.02 (15)	H2A—C2—H2B	109.1
C6—N6—H66	120.4 (19)	C3—N2—H22	119.4 (15)
C6—N6—H6	119.0 (19)	C3—N2—H2	120.6 (19)
H66—N6—H6	117 (3)	H22—N2—H2	120 (2)
O1—C5—N4	126.58 (18)	C3—N3—C4	127.85 (15)
O1—C5—C7	127.43 (17)	C3—N3—C2	110.50 (15)
N4—C5—C7	105.99 (14)	C4—N3—C2	121.64 (15)
N5—C6—N6	126.98 (17)	N3—C3—N2	127.02 (17)
N5—C6—N4	110.34 (15)	N3—C3—N1	110.42 (15)
N6—C6—N4	122.61 (17)	N2—C3—N1	122.56 (17)
N5—C7—C5	102.69 (14)	N3—C4—H4A	109.5
N5—C7—H7A	111.2	N3—C4—H4B	109.5
C5—C7—H7A	111.2	H4A—C4—H4B	109.5
N5—C7—H7B	111.2	N3—C4—H4C	109.5
C5—C7—H7B	111.2	H4A—C4—H4C	109.5
H7A—C7—H7B	109.1	H4B—C4—H4C	109.5
N5—C8—H8A	109.5		
			
Cl1—Cd1—O1—C5	79.99 (16)	O1—C5—C7—N5	179.62 (18)
Cl4—Cd1—O1—C5	−38.13 (16)	N4—C5—C7—N5	−0.20 (19)
Cl2—Cd1—O1—C5	−157.67 (17)	C3—N1—C1—O2	172.47 (17)
Cd1—O1—C5—N4	2.7 (3)	C3—N1—C1—C2	−5.74 (19)
Cd1—O1—C5—C7	−177.09 (13)	O2—C1—C2—N3	−172.51 (18)
C6—N4—C5—O1	178.19 (18)	N1—C1—C2—N3	5.63 (18)
C6—N4—C5—C7	−2.0 (2)	C1—C2—N3—C3	−3.83 (19)
C7—N5—C6—N6	179.08 (18)	C1—C2—N3—C4	177.23 (15)
C8—N5—C6—N6	8.7 (3)	C4—N3—C3—N2	−1.6 (3)
C7—N5—C6—N4	−3.8 (2)	C2—N3—C3—N2	179.59 (18)
C8—N5—C6—N4	−174.16 (17)	C4—N3—C3—N1	179.35 (16)
C5—N4—C6—N5	3.7 (2)	C2—N3—C3—N1	0.5 (2)
C5—N4—C6—N6	−179.05 (17)	C1—N1—C3—N3	3.5 (2)
C6—N5—C7—C5	2.43 (19)	C1—N1—C3—N2	−175.66 (17)
C8—N5—C7—C5	173.30 (16)		

### Hydrogen-bond geometry (Å, °)

**Table:**

*D*—H···*A*	*D*—H	H···*A*	*D*···*A*	*D*—H···*A*
N1—H1···Cl3	0.79 (2)	2.41 (2)	3.1840 (16)	168 (2)
N2—H2···Cl3^i^	0.80 (3)	2.48 (3)	3.273 (2)	170 (3)
N4—H4···Cl4	0.75 (2)	2.70 (2)	3.2967 (17)	139 (2)
N6—H6···Cl2^ii^	0.88 (3)	2.32 (3)	3.1673 (18)	161 (3)
N2—H22···Cl1	0.90 (2)	2.34 (2)	3.2368 (18)	171 (2)
N6—H66···Cl4^iii^	0.81 (3)	2.55 (3)	3.2117 (17)	140 (2)

Symmetry codes:  (i) *x*, *y*+1, *z*; (ii) *x*−1, *y*, *z*; (iii) −*x*−1, −*y*, −*z*+1.
